# Increasing the Stability of Isolated and Dense High-Aspect-Ratio Nanopillars Fabricated Using UV-Nanoimprint Lithography

**DOI:** 10.3390/nano13091556

**Published:** 2023-05-05

**Authors:** Michael J. Haslinger, Oliver S. Maier, Markus Pribyl, Philipp Taus, Sonja Kopp, Heinz D. Wanzenboeck, Kurt Hingerl, Michael M. Muehlberger, Elena Guillén

**Affiliations:** 1 PROFACTOR GmbH, 4407 Steyr-Gleink, Austria; 2 Center for Surface and Nanoanalytics, Johannes Kepler University Linz, 4040 Linz, Austria; kurt.hingerl@jku.at; 3 TU Wien, Institute for Solid State Electronics, 1040 Vienna, Austria

**Keywords:** nanoimprint lithography, nanopattern transfer, aspect ratio, high aspect ratio, nanopillars, simulations for nanoimprinting, nanoneedles, FEBID, black silicon, nanostructuring, nanoelectrode, UV-NIL, nanoscale metal pattern

## Abstract

Structural anti-reflective coating and bactericidal surfaces, as well as many other effects, rely on high-aspect-ratio (HAR) micro- and nanostructures, and thus, are of great interest for a wide range of applications. To date, there is no widespread fabrication of dense or isolated HAR nanopillars based on UV nanoimprint lithography (UV-NIL). In addition, little research on fabricating isolated HAR nanopillars via UV-NIL exists. In this work, we investigated the mastering and replication of HAR nanopillars with the smallest possible diameters for dense and isolated arrangements. For this purpose, a UV-based nanoimprint lithography process was developed. Stability investigations with capillary forces were performed and compared with simulations. Finally, strategies were developed in order to increase the stability of imprinted nanopillars or to convert them into nanoelectrodes. We present UV-NIL replication of pillars with aspect ratios reaching up to 15 with tip diameters down to 35 nm for the first time. We show that the stability could be increased by a factor of 58 when coating them with a 20 nm gold layer and by a factor of 164 when adding an additional 20 nm thick layer of SiN. The coating of the imprints significantly improved the stability of the nanopillars, thus making them interesting for a wide range of applications.

## 1. Introduction

Bioinspired high-aspect-ratio (HAR) micro- and nanostructures are of great interest in nanotechnology today. Many applications benefit from the surface micro- and nanotopographies found in nature. Great examples are the lotus effect, the anti-reflective moth eye, bactericidal effects on, e.g., dragonfly wings, and many more [1,2]. In particular, when it comes to anti-reflection and bactericidal effects, HAR nanostructures, which are nanostructures with a large length-to-diameter ratio, are of great interest [3,4,5]. In technical applications, HAR structures of various sizes are utilized for bactericidal surfaces [6], broadband anti-reflective surfaces [7,8], the modification of stem cells [9,10], in microfluidics [9,11,12], as microneedles [13] or in nanogenerators [14]. In life science applications, well-defined and isolated nanopillars (sometimes called nanoneedles, nanoneedle electrodes or nanoelectrodes if electrically conductive) are of importance, for example, to penetrate cell membranes [15], for intracellular applications or to make recordings [16,17,18].

There is a variety of top-down fabrication methods that are used to realize dense HAR structures in the range from a few tens of nanometres to a few hundred nanometres in diameter. Typical stochastic fabrication methods that are used for dense HAR structures are reactive ion etching (RIE), resulting in so-called black silicon (b-Si) [2]; wet chemical etching processes [19,20]; or laser-induced surface self-organization [21], to name a few. To realize isolated or an array of HAR nanopillars with base diameters below 500 nm, only a few mastering techniques are available. Focused-electron-beam-induced deposition (FEBID) and similar [22,23,24], or two-photon polymerization (2PP) 3D printing [25] can be used. In particular, when it comes to isolated nanopillars with base diameters below 200 nm, FEBID is a capable mastering technique. For all fabrication methods, the achievable aspect ratio and the base and tip diameters are highly dependent on the process parameters and materials. These manufacturing methods typically have significant drawbacks in terms of fabrication time and cost, throughput, materials or suitable substrate sizes, which severely limits the potential for widespread use in mass products.

One of the most versatile nanofabrication techniques today is nanoimprint lithography (NIL). NIL allows for the replication of structures ranging in size from hundreds of micrometres to a few nanometres with nanometre resolution using a physical replication process [26,27,28,29]. NIL was shown to be a cheap and easy fabrication method for a wide range of nanostructures [30,31,32,33], especially in the case of UV-NIL where an imprint resist gets cured by UV radiation.

Over the years, the replication of HAR micro- and nanostructures by NIL was demonstrated [4,34,35,36,37,38]. In general, HAR line and space structures are more stable than HAR pillars, and hot embossing shows better replication results compared with UV-NIL. Aspect ratios of up to ~20 can be achieved for line and space structures by using hot embossing processes [39,40], but in this case, the imprint process is typically more complex and time-consuming compared with UV-NIL. UV-NIL allows for a fast process in ambient conditions and a larger throughput due to the lower-viscosity materials, the faster solidification process, and the lack of heating and cooling steps. With an increased aspect ratio, the specific surface area increases and replication becomes more difficult [41]. For HAR nanostructures, besides the aspect ratio, the tip and base diameters are also of great importance and influence the effects caused by the structures to a great extent. For example, the tip geometry and diameter are strongly influencing bactericidal effects [40]. Furthermore, the mechanical properties dramatically depend on the pillar geometry. In general, the bigger the aspect ratio and the smaller the base diameter of the HAR micro- or nanostructures, the increasingly unstable they become. In the case of bactericidal surfaces, isolated nanopillars or nanoelectrodes, the structure needs to survive certain loads caused by capillary forces when in contact with liquids or the penetration of cells [15]. For large-area optical anti-reflective applications, the structures must withstand certain handling and cleaning processes. When it comes to the replication of HAR nanostructures, NIL faces several challenges with diameters in the range of a few hundred nanometres and below. For successful replication, the structures must survive the separation from the stamp during the imprint process. Separation forces are strongly dependent on the total vertical surface area and the surface roughness, as well as on the materials used and the imprint technology [42,43,44]. Furthermore, stability during fabrication is often a problem, as capillary forces are strong enough to deform HAR nanostructures, which can cause uncontrollable obstacles. There are some techniques where a collapse is intended, such as in capillary-force-induced collapse lithography for the realization of nanogaps for plasmonic nanosensors [45], self-organized arrays [46] or the self-assembly of microstructures [47]. However, for applications where the HAR structures need to be intact and must withstand certain loads, a collapse of the nanostructures is unwanted. Hong et al. presented values for the necessary penetration force of cell walls for algal cells with nanoneedles. The penetration forces depend on the tip diameter and are in the range of ~50 nN for a 100 nm tip diameter, ~90 nN for a 200 nm tip diameter and ~300 nN for a 400 nm tip diameter [15]. Although the bactericidal effect of nanostructures is not yet fully understood, the HAR nanostructures must be able to withstand the rupturing of cell walls [6]. The values given are good indications of the necessary stability.

In this study, the replicability, stability and coating of dense and isolated HAR structures fabricated via UV-NIL was investigated. The tip and base diameters needed to be as small as possible. Isolated FEBID nanopillars and dense black Si substrates were used as masters. Therefore, a FEBID process was developed to deposit single nanopillars and nanopillar arrays. In particular, for the replication of HAR features, the ripping off, incomplete replication, rounding of the tip diameter and the collapse of pillars needed to be avoided during and after the process. The mechanical stability with respect to material parameters was studied using finite element method simulations and buckling analysis in ABAQUS/CAE. We present simulation results and relate them with experiments to give an idea about the stability of such nanopillars made from OrmoComp^®^. In the second step, coating processes to alter the mechanical properties and add electrical functionality were developed. The presented results can lead to the cost-effective and widespread use of HAR nanostructures fabricated via UV-NIL for applications such as anti-reflective coatings, bactericidal surfaces or isolated nanoelectrodes.

## 2. Materials and Methods

### 2.1. Materials

As stamp materials, Sylgard 184 (Biesterfeld Spezialchemie, Austria) and h-PDMS, mixed from Vinylmethylsiloxan, Methylhydrosiloxan (abcr), platinum catalyst and 2,4,6,8-Tetramethyltetravinylcyclotetrasiloxan (Merck), were used. For imprinting, the nanoimprint resist OrmoComp^®^ (OC) (microresist technology, Berlin, Germany), anti-sticking layer BGL-GZ-83 (Profactor GmbH, Steyr-Gleink, Austria) and adhesion promoter HMNP12 (Profactor GmbH, Steyr-Gleink, Austria) were used.

### 2.2. Master Fabrication

**Figure 1:**
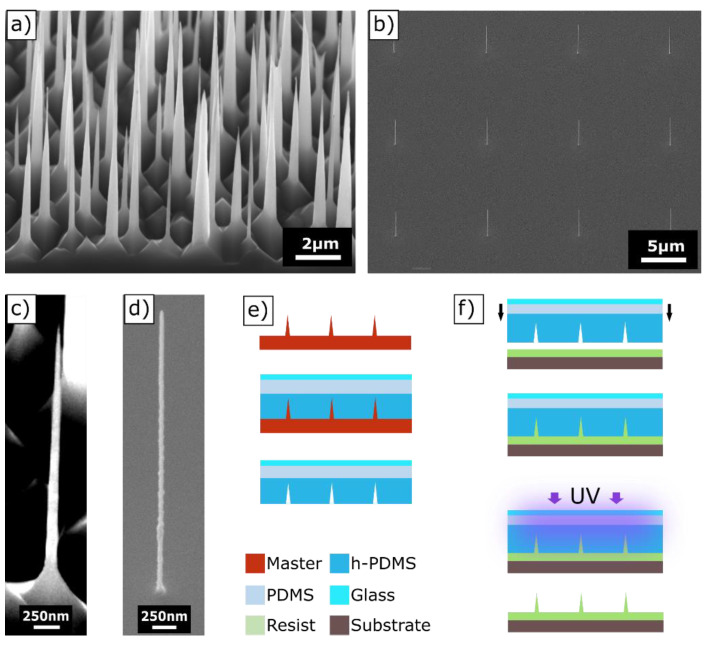
SEM images of dense and isolated HAR nanostructures. Dense nanostructures called black silicon (b-Si) (**a**) and isolated nanopillars made using FEBID deposition (**b**). Close-up of one HAR nanopillar on the b-Si master with a diameter of 250 nm, height of 2.5 µm and aspect ratio of 10 (**c**). Close-up of an HAR FEBID pillar with a diameter of 65.9 nm, height of 3.1 µm and aspect ratio of 47 (**d**). Schematic stamp fabrication process from a master (**e**). Schematics of the general UV-NIL process: First the resist was deposited on a substrate, followed by contacting the stamp with the resist, UV curing and separation (**f**).

In this work, two types of masters (see Figure 1) were used. The first master was fabricated via RIE etching, which resulted in dense HAR nanopillars (obtained from Tekniker [6]). The second master type with the isolated nanopillars was fabricated with a FEBID process. Nanostructures with various shapes and sizes can be fabricated, e.g., HAR nanopillars were reported in [48]. The flexibility of tuning the shape of the depositions and the freedom to place them in an arbitrary position on top of a substrate were the key factors why FEBID was used. For the FEBID deposits, a trimethyl(methylcyclopentadienyl)platinum(IV) (CAS-No.: 94442-22-5) precursor was introduced into an electron microscope (Zeiss Neon 40 EsB Crossbeam SEM-FIB), where it adsorbed onto the substrate [22]. The precursor was exposed to an electron beam and dissociated, which resulted in a solid deposit and volatile reaction products [49]. The beam settings for the used deposits were an extractor voltage of 5 kV with an aperture of 30 µm and a dwell time of 90 s. For imprinting, the FEBID deposits were directly used and some were coated with a 150 nm layer of SiN using plasma-enhanced chemical vapor deposition (PECVD) (Oxford Instruments PlasmaPro 100 PECVD, 300 °C, SiH_4_ 80 sccm, pressure 1.3 × 10^−6^ bar, power 40 W, time 720 s) to increase the stability. All masters were treated with the spin-coated anti-sticking layer BGL-GZ-83 [50].

### 2.3. Stamp Fabrication and UV-NIL Replication

First, stamps were fabricated from the anti-sticking-layer-treated masters similar to the fabrication reported in [51] and depicted in Figure 1e. Therefore, h-PDMS was mixed according to [52], spin-coated onto the masters (2000 rpm for 1 min) to a thickness of ~12 µm and pre-cured for 5 min at 50 °C on a hotplate. Sylgard 184 PDMS was poured on it and spread to a thickness of around 1 mm. Finally, a 300 µm thick glass backplane was placed on top of the liquid PDMS. Afterwards, the layer stack was cured for 12 h at 50 °C on a hotplate.

In the next step, the stamp was used for imprinting in a plate-to-plate process (depicted in Figure 1f). The substrate (Si wafer) was plasma-activated for 2 min at 15 sccm O_2_ and 240 W power (Diener Nano) to enhance the adhesion of the imprint resist. As imprint material, the hybrid organic–inorganic photopolymer OC was used. For imprinting, an in-house-built imprint tool was used, which can be covered by a hood to control the atmosphere (N_2_ or other gases) and pressure. First, OC was deposited on a substrate and the stamp was placed a short distance (~1 mm) above the resist. The hood was then closed, evacuated and filled with N_2_ to return to ambient pressure. The stamp was moved down and brought in contact with the imprint resist, and the distance between the stamp and substrate was set to be approximately 70 µm. No additional forces were applied during the imprint process. After contact, the resist was cured with UV light at a wavelength of 365 nm for one minute with an intensity of 30 mW/cm^2^. The separation between the stamp and resist was manually performed by pulling the stamp after removing the hood and stamp holder.

### 2.4. Characterization

Characterization was performed using scanning electron microscopy (SEM) (Zeiss1540 XB Oberkochen, Germany and SEM, FIB Zeiss NEON 40EsB CrossBeam) and optical microscopy (NIKON, Eclipse LV150N). Contact angle measurements were performed with ethanol, water and liquid gallium (KRÜSS DSA 100). For the ethanol and distilled water, a drop was applied to the sample and a wait time was applied until it evaporated completely. Liquid gallium (heated to 50 °C before usage) was dropped on the samples and then manually removed via vacuuming since evaporation was not feasible.

The profile of the FEBID pillar was derived from the SEM image using ImageJ [53]. First, the gamma of the SEM image was adjusted and converted into an 8-bit black-and-white image. Then, the region of interest was masked, an outline was generated and the x-y values of the outline were saved. These data points were used to calculate the arithmetic roughness Ra, where only the middle 90% of the data set was used to avoid distortion of the measurement by including the tip and the base. The arithmetic roughness was calculated as the area between the profile and the mean of the profile [54].

### 2.5. Post-Processing, Coating and Electrical Functionality

To add electrical functionality, the imprints were sputter-coated with 10 nm Ti and 100 nm Au (VonArdenne LS 320 S). A PECVD-SiN layer was deposited on top of the structures for insulation (Oxford Instruments PlasmaPro 100 PECVD, 250 °C, SiH_4_ 80 sccm, pressure 1.3 × 10^−6^ bar, power 40 W, time 480 s). After SiN coating, the imprints were partially covered via spin-coating AZ 5214 E (MicroChemicals GmbH, Germany) as the photoresist. The photoresist was diluted 1:1 with 1-methoxy-2-propyl-acetate (MicroChemicals GmbH, Germany) and spin-coated with 4500 rpm to only partially cover the nanopillar imprints. The imprints were etched with reactive ion etching (Oxford Instruments—PlasmaLab 100, 180 s, 60 W, 1.31 × 10^−2^ mbar, SF_6_ 50 sccm). 

### 2.6. Simulations

On the basis of the lateral collapse model of Verschuuren [51] (Ch. 2.3.2, p. 20ff) and the experimental observations, a model for the HAR nanopillars was developed. Mechanical stability with respect to the material parameters was studied using finite element method simulations in ABAQUS/CAE. A buckling analysis with different mesh sizes was performed and compared with the analytical Euler buckling force. In a further step, these results were used to perform a post-buckling analysis to gain knowledge about the strain energy with respect to the deflection of the polymeric nanopillar (imprinted nanopillar in OC). Furthermore, the critical buckling force for the coated nanopillars was calculated. Detailed information on the calculation can be found here [55].

## 3. Results and Discussion

### 3.1. Experimental Design

Two types of masters with isolated and densely packed nanopillars with very small base and tip diameters were selected. The master for the dense nanopillars, namely, the so-called black silicone (b-Si) master, was fabricated using a stochastic RIE etching of a silicon wafer (received from Tekniker [56]). SEM images are shown in Figure 1a with a detailed image of a corresponding pillar in Figure 1d. The aspect ratio for the black Si master was not uniform, rather a variety of different aspect ratios was present. Typical dimensions were heights from 3 to 5 µm and base diameters of 150 to 600 nm. The conical pillars on the master had an average tip diameter of 30 ± 13 nm. The aspect ratios ranged from AR 5 < b-Si < AR 20. The aspect ratio shown in Figure 1c is 10.

Isolated nanopillar masters were fabricated using a FEBID process at TU Vienna. The benefit of FEBID nanopillars is that they can be deposited on predefined regions of a substrate with specific geometries. Examples are shown in Figure 1b,d. With the FEBID process, the heights and diameters of the nanopillars could be adjusted depending on the writing time, e-beam focus and movement for each pillar. On the FEBID master aspect ratios up to 47 were realized with base diameters down to 40 nm. The differences between FEBID pillars were the shape of the pillars and their surface quality. The FEBID pillars were cylindrical and not as conical as the b-Si needles. When visually comparing the surface quality of the individual pillars (Figure 1c,d), significant differences between both fabrication techniques became visible. While the surfaces of the b-Si nanopillars appeared flat in the SEM images, they were significantly more rough than the FEBID structures. It is well known that increased roughness and, therefore, increased surface area negatively affect the separation in a UV-NIL process [42]. Additional images of the masters are shown in the Supplemental Materials (Figures S1 and S2).

To develop the UV-NIL process, first, the correct stamp material and stamp design was evaluated. It turned out that the b-Si master was robust and insensitive to the stamp material and the stamp composition. Masters with isolated nanopillars were more fragile and replication was only possible with a SCIL-type stamp to a limited extent [52]. The best results were realized by using a stamp comprising an h-PDMS/PDMS layer stack with a glass backplane. The backplane served as stabilization to prevent distortions; otherwise, ripping off of the isolated nanopillars when removing the stamp could occur. The best replication results were achieved with h-PDMS with a Young’s modulus of 11 MPa. Harder materials, such as X-PDMS, lead to an increased rupture of isolated pillars and lower flexibility of the stamp in our case. The stamp fabrication process is depicted in Figure 1e and the UV-NIL process is shown in Figure 1f. The hybrid photopolymer OC was used as the imprint material for the reasons of thermal stability and biocompatibility [57,58].

**Figure 2:**
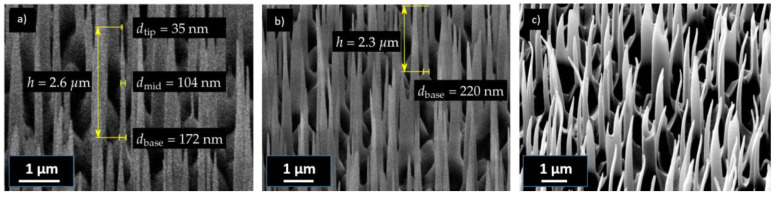
SEM images of replicated uncoated UV-NIL b-Si structures in a photopolymer with an aspect ratio of 15.1 and a tip diameter of 35 nm (**a**) and an aspect ratio of 10.8 (**b**) (acceleration voltage 3 kV). SEM image of sputter-coated b-Si imprints (acceleration voltage 5 kV) (**c**).

The ripping off, rounding of tips, and the bending or collapse of pillars need to be avoided during and after the process. Test imprints were performed in ambient and N_2_ atmospheres, and it was found that the smallest tip diameters could be achieved in the N_2_ environment. According to the manufacturer, replications down to 100 nm should be possible, but since the pillar tips were significantly smaller, some inhibition with the crosslinking could be assumed. In order to inspect the smallest achievable tip diameter, imprinting was performed in a N_2_ atmosphere in an in-house-built imprint tool. Of course, this effort is not necessary for applications where pillars with larger tip diameters are acceptable. HAR structures of up to 15.1 with a tip diameter of 35 nm from the dense b-Si master could be replicated, as shown in Figure 2a,b. As can be seen from the SEM images in Figure S3 and Video S1 in the Supplementary Materials, during the inspection, the electron beam led to a strong deformation of the pillars within a few seconds if the pillars were not sputter-coated. The sputter-coating greatly prevented deformation during the inspection. In the case of a metal sputter-coating before SEM inspection, the images had a better contrast, as it was possible to work with a higher acceleration voltage (Figure 2c). However, the coating already changed the tip geometry of the pillars and led to a rounding [38]. In the metal-sputter-coated samples (Figure 2c), the tip diameter was 61.3 ± 15 nm. In order to avoid distortion and bending of the nanostructures and to be able to measure the true tip shape and diameter of the replicated pillars, SEM inspections needed to be performed on uncoated samples. The tip diameter for uncoated pillars found by measuring only the sharp tips from Figure 2a was 38.9 ± 13 nm. This shows that the replication fidelity for the imprinting process itself could be very good.

In the next step, replication experiments with FEBID masters were performed. Therefore, several masters with different pillar geometries were fabricated using FEBID with diameters ranging from 36 nm to 300 nm and corresponding heights to a few µm (Figure 1). The FEBID process was well suited to make arrays or individual HAR nanopillars with aspect ratios up to 83.

**Figure 3:**
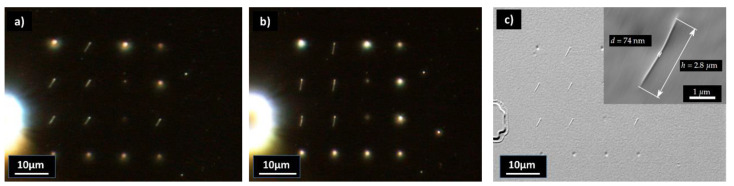
Replication results of pillars with an aspect ratio of >37. Dark-field image of two different imprints with collapsed needles (**a**,**b**). SEM image of the sample in (**a**) is shown in (**c**). The insert in (**c**) shows a detail of a pillar with a 74 nm diameter and a length of 2.8 µm.

In Figure 3, imprinted FEBID pillars with aspect ratios >37 are shown. The pillars collapsed after or during the separation, suggesting that the polymer structures were not stable enough to allow for free-standing pillars. The pillars collapsed in different directions, as shown in Figure 3a,b, indicating that the replication process and the separation from the stamp were successful. In the case where the pillars would have already collapsed on the master, the direction of the pillars must be identical for both imprints. An SEM image of the original master is shown in Figure 1b.

**Figure 4:**
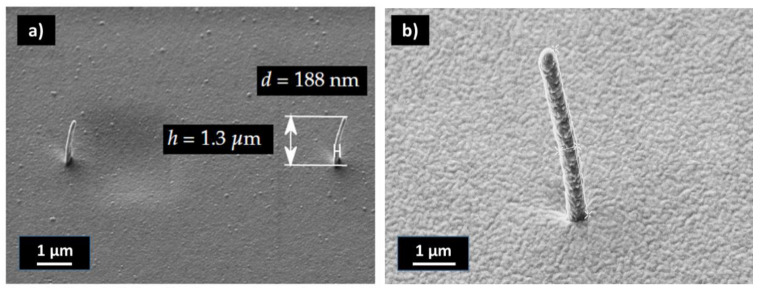
Replicated isolated FEBID nanopillar with an aspect ratio of 6.9, base diameter of 188 nm and tip diameter of 109 nm (**a**). After PECVD coating with SiN of the FEBID master, aspect ratios of up to 8.3 could be achieved with a base diameter of 455 nm and a tip diameter of 409 nm (**b**).

We find that for the use of a master for a UV-NIL fabrication process, FEBID pillars only displayed limited usability. For many FEBID masters, no stamp fabrication was possible in the first place, as a rupture of the pillars often occurred. Due to the destruction of the master, no systematic process optimization was possible. The highest aspect ratio of isolated pillars with the smallest base diameter we could achieve from the FEBID masters was 6.9 with a base diameter of 187.5 nm and a tip diameter of 109 nm (Figure 4a). We assume that the poorer imprint ability of the FEBID masters arose from the surface quality, as the pillars were significantly rougher and less uniform than in the case of the b-Si master (see Figure 1 for comparison). For a quantitative assessment of the surface, the arithmetic roughness of the profile was calculated. The arithmetic mean roughness (Ra) for the FEBID pillar in Figure S4 was calculated to be 1.51 nm, compared with 1.06 nm for the b-Si nanopillars. The maximum peak heights (Rq) were 9.84 nm and 5.15 nm for the FEBID and b-Si nanopillars, respectively. Considering that the diameter in the FEBID nanopillar was only ~40 nm, the Rq corresponded to ~12.8% of the total diameter for the FEBID nanopillar but only ~1.9% for the b-Si pillar. In order to increase the stability of the FEBID master, a 150 nm PECVD-SiN coating of the FEBID pillars was performed. No investigation was made into how thin this coating can be, as no systematic investigation was possible because each master had slightly different surface properties. With the PECVD-SiN coating, aspect ratios of up to 8.3 could be achieved with a base diameter of 455 nm and a tip diameter of 409 nm (Figure 4b).

However, our results clearly showed that with the achievable FEBID pillar stability, the shape and surface quality for nanopillars with base diameters below 180 nm were not sufficient. Often stamp fabrication was not possible in the first place or the replicated pillars were not stable enough.

### 3.2. Stability Considerations and the Influence of Liquids on Dense Polymeric Nanostructures

To investigate the stability of the nanostructures and their interactions with liquids, systematic experiments were performed on the b-Si samples. The stability is dependent on many factors, where not only the size, shape and aspect ratio but also the material parameters, such as the Young’s modulus, play an important role. To this end, a sample of the black silicon master and an imprinted sample were investigated. The selected liquids ethanol, water and gallium differ strongly in their surface tension and, therefore, in the formation of the contact angle and the wetting on the samples. For ethanol and distilled water, a drop was applied to the sample and a waiting period was applied until it evaporated completely at ambient conditions. The measurement of the contact angle of ethanol was not possible (for all samples), as ethanol immediately spread over the surface. This behaviour indicated a very high wetting ability on all samples. No change in the cured OC was expected from the fluids used. For all experiments, the contact angles of the liquids on the samples were determined and are given in Table 1.

**Figure 5:**
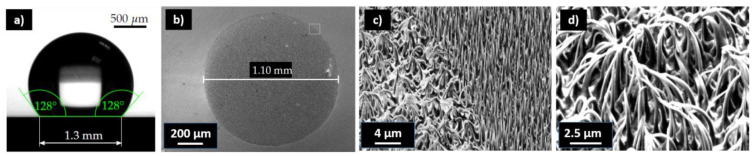
Contact angle measurement of a water drop on an imprinted sample (**a**). SEM images of the area where the evaporating water drop caused a collapse of the pillars on a fresh nanoimprinted sample (**b**). The edge region indicates the pinning of the water drop (**c**). Detailed view of collapsed pillars due to surface tension (samples were metallized for SEM measurements) (**d**).

In general, it can be noted that the contact angle on all surfaces increased when the surface tension of the liquid increased (see Figures S5 and S6 in the Supplementary Materials). However, it can be observed that the wetting experiments had a strong influence on the stability of the nanostructures consisting of different materials with a highly varying Young’s modulus: OC with 1°GPa [60] and Si with 165°GPa. This shows that Si was much stiffer than OC. The following observations could be made for the sample consisting of silicon (b-Si master): The nanostructures on the sample were not permanently affected by any of the liquids. It was not possible to determine whether the nanopillars were deformed and subsequently came into contact with other nanopillars. However, if this happened, it can be assumed that the strain energy of the pillar was greater than the adhesion energy between the pillars, which allowed the pillars to realign after the liquids evaporated. The sample consisting of OC showed a clear interaction between the nanopillars and the liquids ethanol and water. In the areas where the liquid was in contact with the sample surface, the nanopillars were deformed and attached to the neighbouring pillars (Figure 5d). There is a sharp edge visible on the samples where water was dispensed, indicating the pinning of the water drop. Experiments were performed where the samples were frozen right after the application of water or ethanol and, in a second step, the frozen liquid was removed via freeze-drying. In this setting, no pillars collapsed, indicating that the collapse of the pillars occurred during the evaporation of the liquids and not during the application.

**Figure 6:**
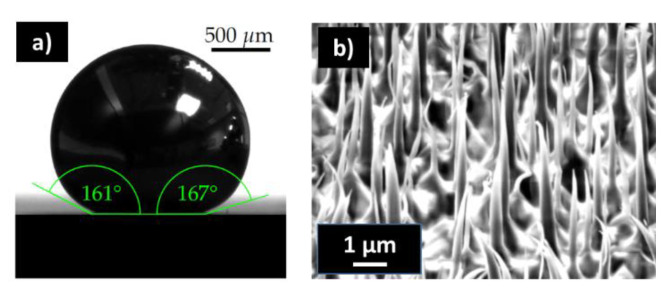
Contact angle measurements for gallium on imprinted samples (**a**). Gallium did not cause a collapse of the pillars, but bent and compressed some pillars close to the tips (**b**).

For gallium, no collapse of nanopillars could be observed on the b-Si master and the imprint. In Figure 6, the contact angle measurements for gallium on an imprinted sample are shown. Although the density of gallium is 5.9 times higher than that of water, gallium did not cause a collapse of the pillars. However, bending and compression close to the tips of some pillars were visible. Again, this supports the observation that the deposition of a liquid did not cause the collapse. In terms of wetting of the liquids on the nanopillar surfaces, ethanol and water showed Wenzel state wetting and gallium showed a Cassie–Baxter-type wetting.

It was observed that ageing of the samples by storing them on the shelf for weeks led to a change in the stability of the nanopillars on the b-Si imprints and allowed them to survive the capillary forces of water and ethanol. This indicated that the material itself was changing over time, which suggested a post-polymerization. This effect was not further investigated, as in a typical fabrication process, the necessity to store samples for some time would be a disadvantage. Assuming that the needles only had full stability after this post-polymerization process, a value can be obtained for the minimum stability of the pillars using the dimensions and material properties.

**Figure 7:**
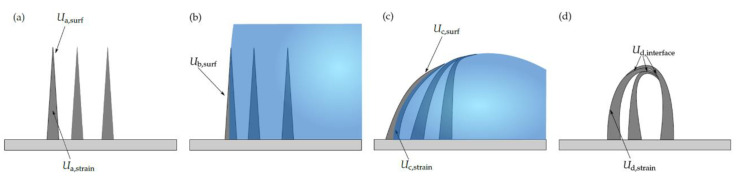
Different states of wetting polymeric nanopillars using liquids. Untreated nanopillars (**a**). Part of a droplet that partially wets the surface and pins at the left pillar (**b**). As the droplet evaporates the surface shrinks and interacts with the pillar, which then comes in contact with another one (**c**). After complete evaporation of the liquid, the energy balance of the interfacial energy between the pillars and the appearing strain energy results in collapsed pillars (**d**).

In the next step, we calculated the stability of the polymeric OC pillars and the expected effect of a coating process. A bending condition was derived, which could describe an approximation of the surface tension between the liquid and the polymer. The energy difference ${∆G}_{ca}$ between the initial state (Figure 7a) and the bent state Figure 7c could be described using

$$\begin{array}{cc} {∆G}_{ca}=G_{c}-G_{a} & =\left(U_{c,strain}+U_{c,surf}\right)-\left(U_{a,strain}+U_{a,surf}\right)\\ & =\left(U_{c,strain}+\gamma_{SG}A_{shell}c_{c}+\gamma_{SL}A_{shell}\left(1-c_{c}\right)\right)-\left(0+\gamma_{SG}A_{shell}\right)\\ & =U_{c,strain}+\gamma_{SG}A_{shell}c_{c}+\gamma_{SL}A_{shell}\left(1-c_{c}\right)-\gamma_{SG}A_{shell}\\ & =U_{c,strain}+A_{shell}\left(1-c_{c}\right)(\gamma_{SL}-\gamma_{SG}) \end{array}$$

where $G_{c}$ is the energy in state (*c*), $G_{a}$ is the total initial energy (*a*), *U_c,strain_* is the stored strain energy, $U_{c,surf}$ is the surface energy, the parameter *c_c_* was introduced and describes the fraction of the shell surface $A_{shell}$ that is wetted by the droplet, and $\gamma_{SL}$ and $\gamma_{SG}$ are the surface energies. Restoration of the pillars occurs if ${∆G}_{ca}$ is positive and bending occurs if ${∆G}_{ca}$ is negative. As the droplet begins to evaporate, the volume shrinks and interacts with the pillar in such a way that the pillar is bent and a strain energy $U_{c,strain}$ appears, as shown in Figure 7c. This phenomenon was already investigated and was described by Neukirch et al. [61].

Further, a collapse condition was derived that confirmed the experimental results: once the pillars came into contact, the occurring strain energy was not sufficiently high to restore the pillars.

$$\begin{array}{cc} {∆G}_{da}=G_{d}-G_{a} & =\left(U_{d,strain}+(U_{a,surf}-U_{d,interface}\right)-\left(U_{a,strain}+U_{a,surf}\right)\\ & =\left(U_{d,strain}+\gamma_{SG}A_{shell}\left(1-c_{d}\right)\right)-\left(0+\gamma_{SG}A_{shell}\right)\\ & =U_{d,strain}+\gamma_{SG}{(A}_{shell}-A_{interface})-\gamma_{SG}A_{shell}\\ & =U_{d,strain}-\gamma_{SG}A_{interface} \end{array}$$

**Figure 8:**
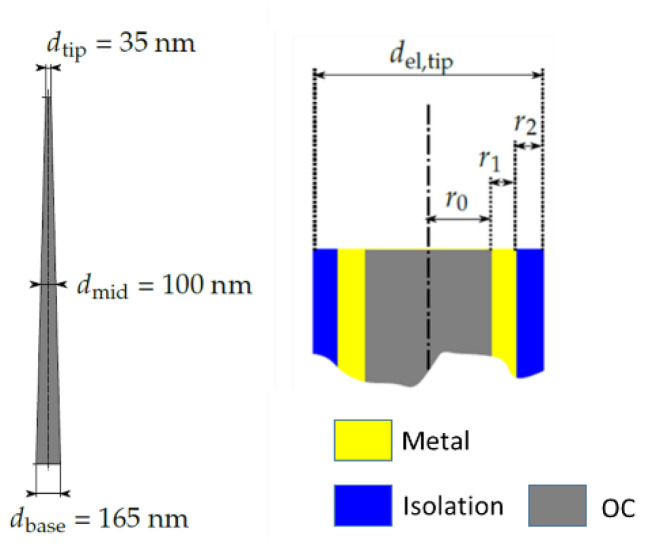
Dimensions of a replicated nanopillar as presented above in Figure 2b. Schematic cross-section of the coated pillars after selective etching of the tip. The different colours represent different material layers and the dimensions represent the layer thicknesses.

If $U_{d,stain}>\gamma_{SG}A_{interface}$, the energy difference ${∆G}_{da}$ is positive, the total energy of the system increases and, therefore, restoration of the nanopillars is favourable and the system returns to the state shown in Figure 7a. If $U_{d,strain}<\gamma_{SG}A_{interface}$, the energy difference ${∆G}_{da}$ is negative, the total energy of the system decreases and the case shown in Figure 7d occurs. Regarding this condition, it is possible to predict the stability of the nanostructures when it comes to bending due to an interaction with the surface of a liquid or adhesion between the nanopillars. The strain energies were determined via simulations using the finite element method on pillar dimensions shown in Figure 8. Further information regarding the buckling and strain energies is shown in Figures S7 and S8 in the Supplementary Materials. Finally, again using the finite element method, the critical force for the nanopillar was calculated for stability reasons. Furthermore, by adding additional layers, the change in critical force was calculated for pillars with an aspect ratio of 15.1.

As shown in Table 2, the critical buckling force (F_crit,buckling_) increased considerably when the imprints were coated with metal or SiN. The stability of a polymeric pillar with a radius of r_mid_ = 50 nm increased by a factor of 58 when coated with a 20 nm gold layer compared with a polymer pillar with r = (r_mid_ + r_1_) = 70 nm. When adding an additional 20 nm thick layer of SiN, the stability increased by a factor of 164.38 compared with an r = 90 nm polymer pillar. By comparing the F_crit,buckling_ values with values from the literature, one sees that the pillars should be stable enough to penetrate a cell membrane [15]. The value for the penetration force for a pillar with a tip diameter of 200 nm was around 100 nN. Our calculation showed that one polymer needle would not be stable enough to survive such a load; however with a 20 nm Au coating, the F_crit,buckling_ was 5 times higher, and when adding 20 nm Au and 20 nm SiN, the F_crit,buckling_ was 42 times higher. Furthermore, a collapse of the pillars should not be observable after coating (metal and isolation coating) anymore as the stability of the needles increased. By comparing the F_crit,buckling_ force with the force necessary for cell wall penetration, it became obvious that only coated nanopillars can be stable enough to accomplish this. A nanopillar with a 105 nm tip diameter made of OC could withstand 26 nN, while a nanopillar with Au and SiN coating with the same tip diameter could withstand 4274 nN. For comparison, the loads were in the range of ~50 nN for 100 nm tip diameter, as reported in [15].

### 3.3. Coating of Nanoimprinted Nanostructures

Based on the calculations to increase the stability of the pillars, a coating strategy was developed that worked on replicated polymer nanopillars (b-Si and FEBID), as well as on master structures (FEBID pillars). The samples were sputter-coated with 10 nm Ti and 100 nm Au, followed by the deposition of a PECVD-SiN layer for insulation. The Au layer was very thick to achieve a sufficient thickness on the sidewalls of the pillars. The high temperature of 300 °C, which is required for the PECVD process, was no problem for the metal-coated OC imprints.

**Figure 9:**
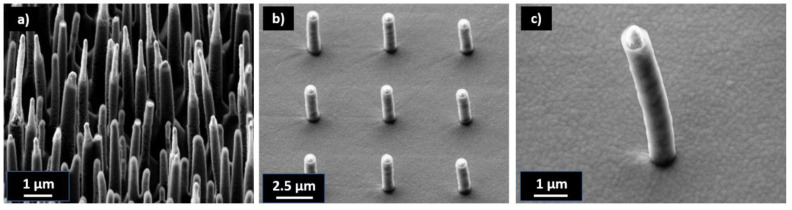
Black silicon imprints after coating and SiN etching. Only the uncoated areas on the tips were etched with the RIE process (**a**). An array of isolated nanopillars replicated from a FEBID master after coating and after SiN etching with open tips. The aspect ratio of the imprints was 7 (**b**). Detail on coated pillars after removing the isolation at the tip (**c**). The imprint from the FEBID master is shown in Figure 4b.

In order to realize nanoelectrodes, it is necessary to remove the SiN insulation from the tips by performing a selective etching step. Therefore, after the SiN coating, the imprints were partially covered via spin-coating with AZ 5214 E. The samples were then etched with reactive ion etching, followed by stripping of the photoresist. With this, the metal was not isolated anymore and the pillars were electrically conductive at the tip and the pillars were transformed into nanoelectrodes. The etching of the SiN layer on the dense and isolated pillar arrays is shown in Figure 9. The average tip diameter for the dense nanopillars in Figure 9a with metal and SiN coating was 180.6 ± 17 nm, and with only metal coating, it was 101.2 ± 21 nm. No collapse of pillars could be observed anymore after coating the b-Si samples, as was expected.

An array of isolated nanopillars is shown in Figure 9b and details of a pillar after coating and tip opening are shown in Figure 9c. The base diameter after coating for the pillar in Figure 9c was 874 nm with a height of 3.8 µm. An SEM image of the same pillar before the coating is shown in Figure 4b. By comparing the tip diameters for both needles (tip diameter was 475 nm after metal coating), one sees that a metal layer of 30 nm was deposited with the selected settings on the sidewalls of the pillars. Therefore, good electrical properties of the pillars were expected. Electrical characterization of the nanoelectrodes is planned and will be presented elsewhere.

## 4. Conclusions

A process for the replication of HAR nanopillars using UV-NIL was proposed and compared for different masters. In addition, a coating process was developed to increase the stability and functionality of the replicated HAR nanopillars.

For the first time (to the best of our knowledge), dense HAR nanopillars made from a b-Si master with an aspect ratio of 15 and a tip diameter of 35 nm were fabricated using a UV-NIL process. An aspect ratio of 8.9 was obtained for isolated HAR nanopillars from FEBID masters. Interestingly, the replication of isolated HAR nanopillars with an aspect ratio of 37 and a base diameter of 75 nm could be demonstrated. However, free-standing nanopillars with these dimensions could not be observed.

Stability investigations revealed that imprinted HAR nanopillars (b-Si) made of OrmoComp^®^ were not stable enough to withstand the capillary forces that occurred during the evaporation of water or ethanol, resulting in a collapse of the nanopillars. However, the Si master remained unchanged with the same loads. Stability simulations of imprinted nanopillars were performed and the change in stability when adding Au or SiN coating was calculated. The stability of a pillar could be increased by a factor of 58 when coating with a 20 nm gold layer and by a factor of 164 when adding an additional 20 nm thick layer of SiN. A coating process based on sputtering and PECVD deposition was demonstrated for imprinted samples, as well as for FEBID masters. After the coating processes, the nanopillars were stable enough to survive capillary forces for any wet chemical treatment. In the final process, the isolation layer could be selectively etched on the needle tips, which made them potential nanopillar electrodes.

With the presented UV replication process, the replication and the subsequent stability increased due to the coating processes; as a result, the HAR nanopillars become interesting for a wide range of applications, from large-area structural anti-reflective coatings to bactericidal surfaces. When applying the additional RIE step to remove the isolation on the nanopillar tips, applications such as nanopillar electrodes are possible.

## Figures and Tables

**Table 1:** Overview of different surface energies of the used liquids and their contact angles on the samples.

Liquid	Surface Tension g_LG_ in mJ/m^2^	Temperature in °C	Contact Angle on b-Si Master	Contact Angle b-Si Imprint	Contact Angle Flat OC
Ethanol	22.27	20	n.a.	n.a.	n.a.
Distilled water	72.75	20	125°	128°	107°
Gallium	708.67 [59]	50	147°	161–167°	150°

**Table 2:** Critical loads of conical nanopillars with and without additional (coating) layers. As the composition changed, the overall radius of the pillar changes as well, whereas the height h = 2.5 µm remained unchanged.

Composite Structure	r_mid_ (nm)	r_1_ (nm)	r_2_ (nm)	d_el,mid_ (nm)	d_el,tip_ (nm)	F_crit, buckling_ (nN)
OC	50			100	35	1.70
OC + OC	50	20		140	75	9.2
OC + OC + OC	50	20	20	180	105	26
OC + Au	50	20		140	75	534
OC + Au + Si_3_N_4_	50	20	20	180	105	4274
Au	50			100	35	132
Au + Au	50	20		140	75	718
Si_3_N_4_	50			100	35	463
Si_3_N_4_ + Si_3_N_4_ + Si_3_N_4_	50	20	20	180	105	7127

## Data Availability

Not applicable.

## Supplementary Materials

The following supporting information can be downloaded from https://www.mdpi.com/article/10.3390/nano13091556/s1—Figure S1: Example of a black silicon master with dense nanoneedles. Figure S2: Example of a typical FEBID master. Figure S3: Influence of the SEM characterization on a b-Si imprint in OC. Figure S4: Calculation of the pillar roughness. Figure S5: Contact angle measurement of water on three different surfaces. Figure S6: Contact angle measurement of gallium on three different surfaces. Figure S7: Buckling modes of the conical polymeric pillars. Figure S8: Bending of a polymeric conical pillar. Video S1: Influence of the SEM characterization on a b-Si imprint in OC.
