# Supplementary material for: Increasing the Stability of Isolated and Dense High-Aspect-Ratio Nanopillars Fabricated Using UV-Nanoimprint Lithography
Source: Nanomaterials (Basel). 2023 May 5;13(9):1556. doi: 10.3390/nano13091556 (PMC10180511; doi:10.3390/nano13091556)
Supplement: Supplementary file 1 [file nanomaterials-13-01556-s001.zip › nanomaterials-Supplementary Materials Increasing stability of Isolated and Dense _review.pdf]

Supplementary Materials

Increasing the Stability of Isolated and Dense High-Aspect-Ratio Nanopillars Fabricated Using UV-Nanoimprint Lithography

M.J. Haslinger[1,*], O. S. Maier[1,2], M. Pribyl[3], P. Taus[3], S. Kopp[1], H.D. Wanzenboeck[3], K. Hingerl[2], M. M. Muehlberger[1], E. Guillén[1]

[1] PROFACTOR GmbH, Steyr-Gleink, 4407, Austria
[2] Center for Surface and Nanoanalytics, Johannes Kepler University Linz, 4040 Linz, Austria
[3] TU Wien, Institute for Solid State Electronics, Vienna, 1040, Austria
* Correspondence: Michael.Haslinger@Profactor.at ; Tel.: +43-7252-885-422

Black silicon master with dense nanopillars:

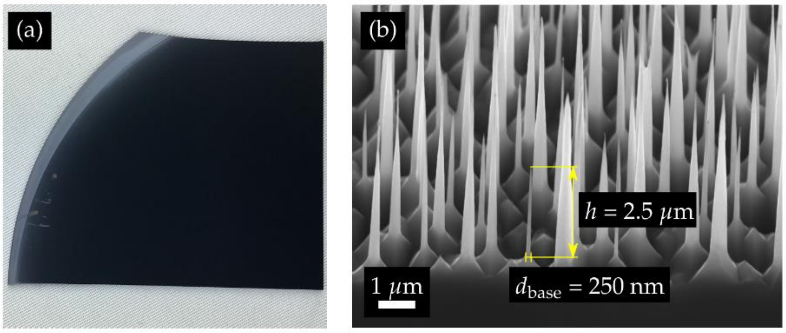

Figure S1 Example of a black silicon master with dense nanoneedles (received from Tekniker [6]) (a). SEM image of nanoneedles with different aspect-rations (b).

FEBID master with isolated nanopillars:

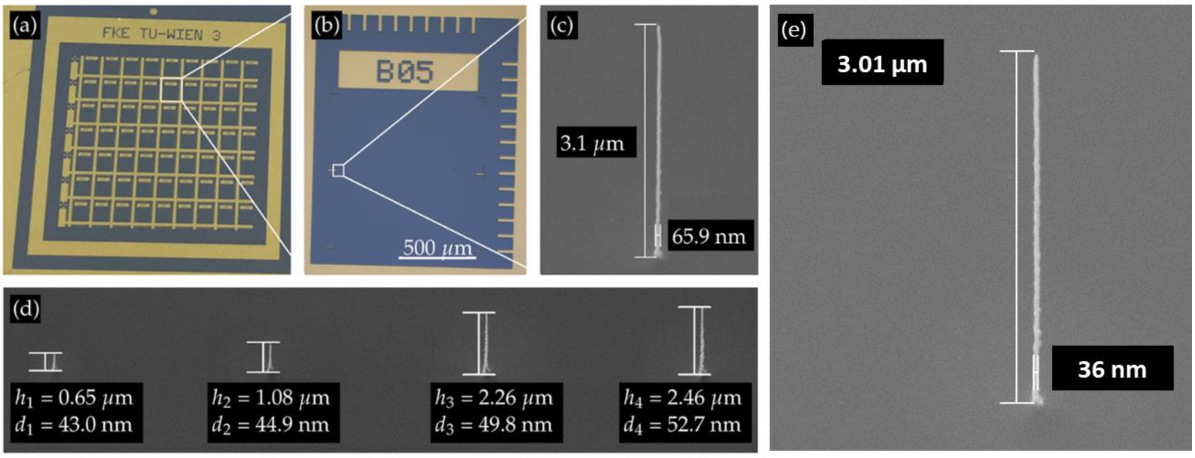

Figure S2 Example of a typical FEBID master. Photograph with the orientation grid (a). Microscope images of a sample with FEBID needles (b). SEM images of nanoneedles with different aspect-rations (c, d, e).

Effect of SEM characterisation on polymeric HAR nanopillars:

Characterization of polymeric high AR needles leads to a change in the shape of the pillars within seconds. Especially in pillar tip diameter characterization the scanning of the electron beam immediately changes the shape of the pillar. To this end, great care had to be taken during characterization in order not to alter the results by excess beam exposure.

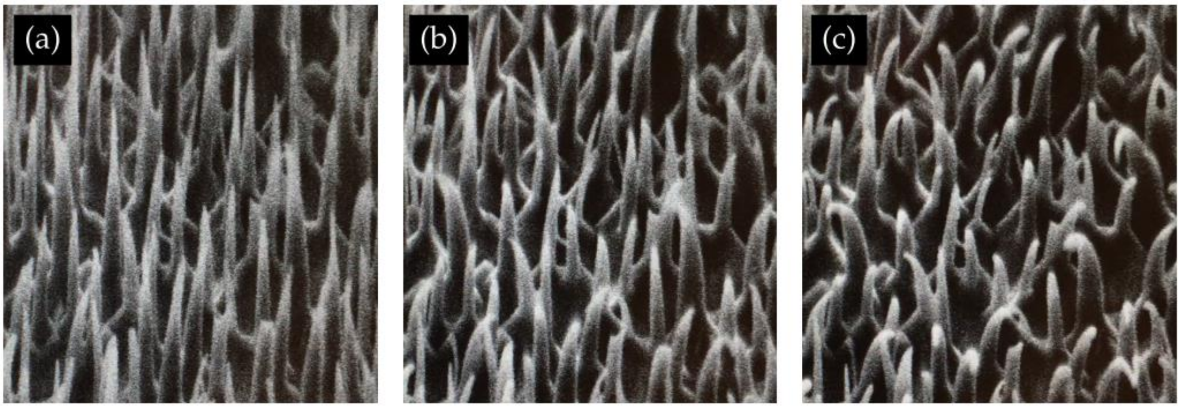

Figure S3 Influence of the SEM characterization on a b-Si imprint in OC. The acceleration voltage was set to 3 kV. Nanostructures directly after focusing (a). Same position, five seconds of continuous scanning after focusing (b). Ten seconds of continuous scanning after focusing (c). Clearly a deformation of the pillars occurs. Even after 5 sec the tip diameters have already changed significantly.

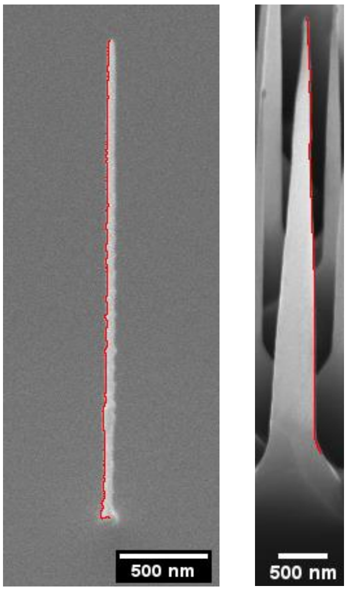

Figure S4 FEBID pillar with profile curve (in red) for calculation of the roughness (left).b-Si pillar with profile curve for roughness calculation (right).

Results on contact angle measurements:

Contact angle measurement of water:

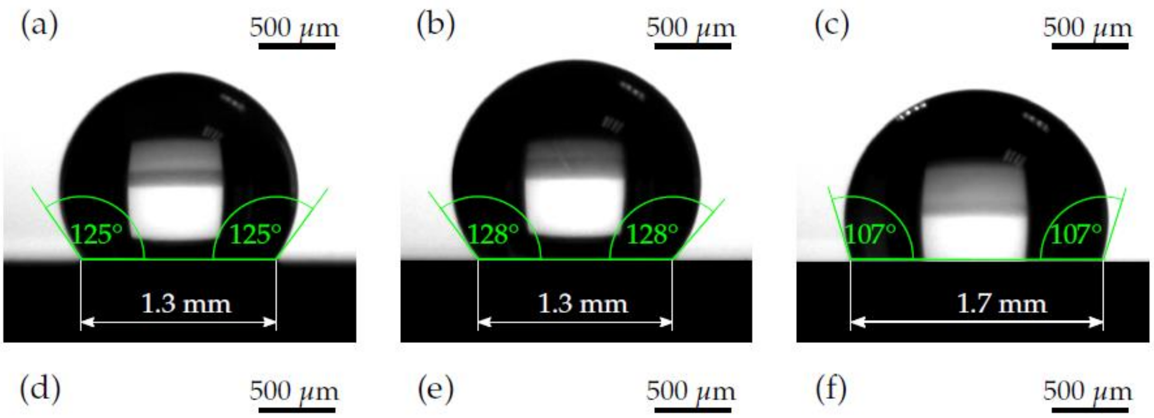

Figure S5 Contact angle measurement of water on three different surfaces. Waterdrop on b-Si master (a), Water on OC b-Si (b) and water on flat OC (c).

Ethanol:

If ethanol is dropped on the samples, it immediately spreads over the entire surface, so a contact angle measurement is not possible here. This behaviour indicates a very high wetting ability of ethanol on all three samples.

Gallium:

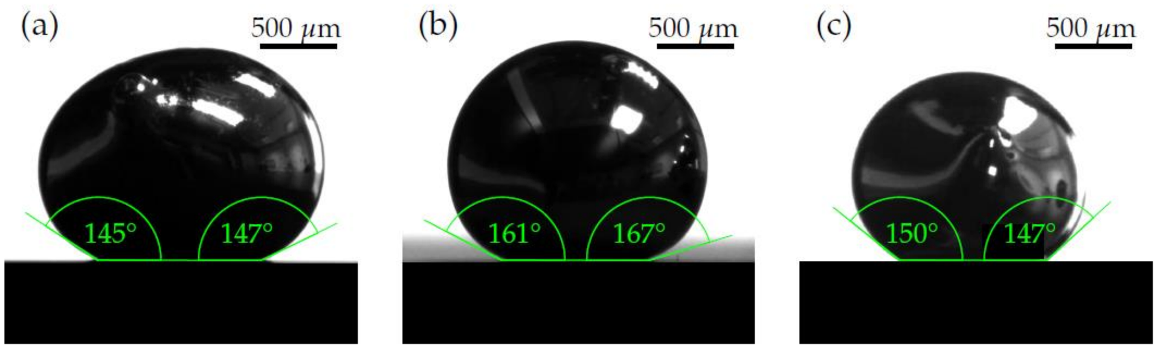

Figure S6: Contact angle measurement of gallium on three different surfaces. Gallium on silicon pillars(a), gallium on OC b-Si (b) and gallium on flat OC.

Buckling modes and critical force depending on simulation parameter:

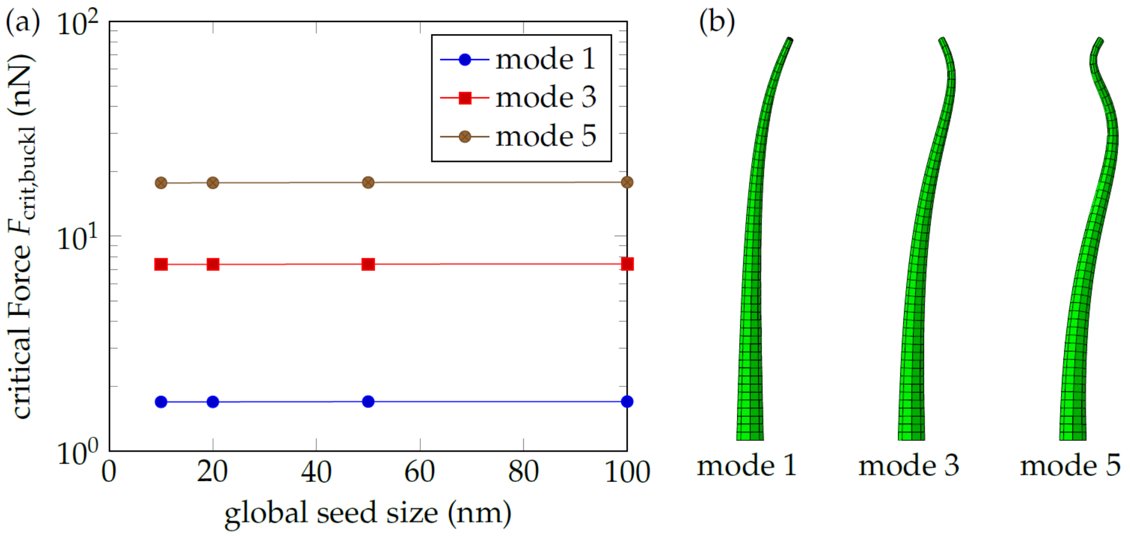

Figure S7 Buckling modes of the conical polymeric pillars. Critical force F_{crit} vs. Global seed size (a). The eigenvalues change when the element size changes. First three modes of the buckling analysis, with a global seed size of 50 nm (b).

Strain energy for conical nanopillars:

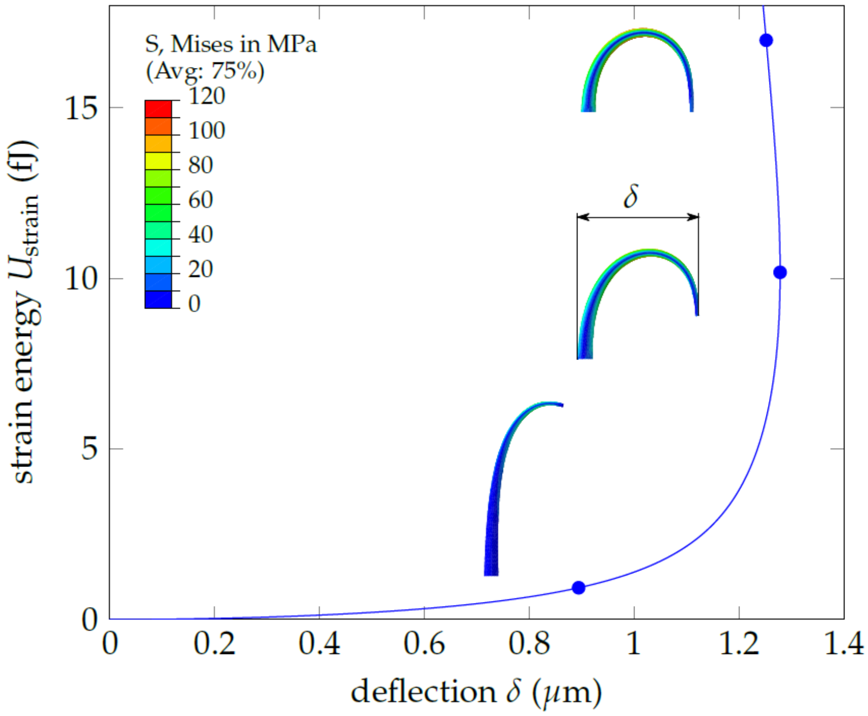

Figure S8 Bending of a polymeric conical pillar. The strain energy U_{strain} increases with rising deflection d. The simulation is performed until the tip reaches the surface. The color legend corresponds with the arising stress inside the pillar.
